# Nanometric holograms based on a topological insulator material

**DOI:** 10.1038/ncomms15354

**Published:** 2017-05-18

**Authors:** Zengji Yue, Gaolei Xue, Juan Liu, Yongtian Wang, Min Gu

**Affiliations:** 1 Laboratory of Artificial-Intelligence Nanophotonics and CUDOS (Centre for Ultrahigh bandwidth Devices for Optical Systems), School of Science, RMIT University, Melbourne, Victoria 3001, Australia; 2 Beijing Engineering Research Centre for Mixed Reality and Advanced Display, School of Optoelectronics, Beijing Institute of Technology, Beijing 100081, China

## Abstract

Holography has extremely extensive applications in conventional optical instruments spanning optical microscopy and imaging, three-dimensional displays and metrology. To integrate holography with modern low-dimensional electronic devices, holograms need to be thinned to a nanometric scale. However, to keep a pronounced phase shift modulation, the thickness of holograms has been generally limited to the optical wavelength scale, which hinders their integration with ultrathin electronic devices. Here, we break this limit and achieve 60 nm holograms using a topological insulator material. We discover that nanometric topological insulator thin films act as an intrinsic optical resonant cavity due to the unequal refractive indices in their metallic surfaces and bulk. The resonant cavity leads to enhancement of phase shifts and thus the holographic imaging. Our work paves a way towards integrating holography with flat electronic devices for optical imaging, data storage and information security.

## Abstract (web summary)

Holograms generally need to be as thick as a wavelength of light to introduce the necessary optical phase shifts that create true three-dimensional images. Here, Yue *et al*. use a high-index topological insulator material to create a resonant optical cavity and thin holograms to the nanometre scale.

Holography has been widely used in optical microscopy, imaging, data storage, metrology as well as information security since it was invented by Dennis Gabor in 1940s (refs 1, 2, 3). The rapid developments of laser and computer technologies leaded to the advent of computer-generated holography (CGH) in 1960s (ref. 4). CGH holds a distinct superiority over the conventional optical holograms because real objects are not required for holographic imaging. It enables generating holographic interference patterns of virtual objects by digitally computing optical wavefronts^5^. With this advantage, CGH has a great potential to be integrated with modern electronic devices, such as smartphone and smart watch for imaging and display^6^. To realize these devices, holograms are required to be thinned to a nanometre scale. However, conventional holograms modulate the phase of light based on the propagation retardation in three-dimensional (3D) bulky materials. The phase modulation capability relies on the propagating distance through the materials, which requires the thickness of holograms within optical wavelength ranges. This drawback impedes the development of nanometric holograms and their integration with modern electronic devices.

The recent development of emerging flat optics demonstrates the potential to break the thickness limit^7^,^8^,^9^. Ultrathin holograms have been designed using various metasurfaces that consist of nanostructure arrays with alterable geometric parameters^3^,^10^,^11^,^12^,^13^. However, limited by the fundamental mechanism of Mie resonances (scattering), dielectric metasurface holograms are unlikely to reach a deep sub-wavelength scale (much less than the optical wavelength). Moreover, the nanofabrication of metasurface holograms requires quite complex facilities, such as focused ion beam lithography and electron-beam lithography. In addition, using the complex focused ion beam lithography or electron-beam lithography techniques, it is also hard to realize large area holograms for practical applications.

Two-dimensional (2D) materials that have attracted a great attention nowadays can provide an excellent platform for realizing ultrathin optical devices^14^,^15^. Topological insulators are unique quantum materials that have topologically protected 1D edge states on 2D insulating bulk states, or 2D metallic surface states on 3D insulating bulk states^16^,^17^. They demonstrate a number of fascinating electronic and optical properties, such as spin-momentum locking, near-infrared transparency and ultra-broadband plasmon excitations^18^,^19^,^20^. It has also been discovered that topological insulator materials hold the low refractive index in the surface layer but the ultrahigh refractive index in the bulk^21^. This unique property makes them promising for designing novel optoelectronic devices including nanometric holograms.

Here, we propose the concept and the experimental realization of nanometric holograms using a topological insulator material Sb_2_Te_3_. We observe that the Sb_2_Te_3_ thin film holds unequal refractive indices in the surface layer and bulk, which makes the thin film acting as an intrinsic resonant cavity. The optical path length and phase shifts of an output light beam from the cavity can be enhanced through internal multi-reflections. Based on this new mechanism, we achieve 60 nm binary holograms of 3 mm × 3 mm in size using a simple and fast direct laser writing (DLW) system^2^. Holographic images are successfully realized with the nanometric holograms.

## Results

### Design of nanometric holograms

**Figure 1:**
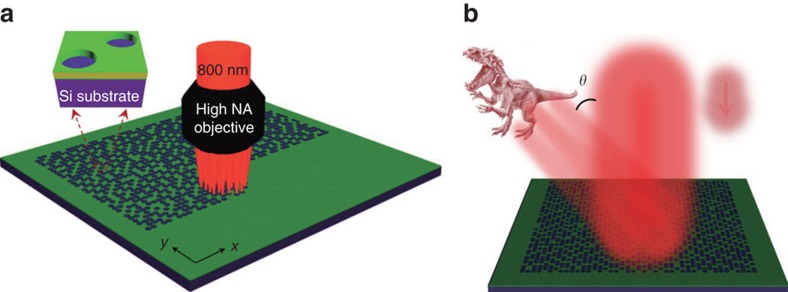
Nanofabrication of the binary hologram and holographic imaging procedures. (**a**) Nanofabrication process of a nanometric Sb_2_Te_3_ hologram using the direct laser write system. The upper inset displays the ablated pixels in the topological insulator thin film. (**b**) Holographic imaging procedure of a nanometric Sb_2_Te_3_ hologram using a continuous wavelaser beam. *θ* is the projection angle of the holographic image. Note: this figure is not included under the article CC BY licence; Indominus Rex image is reproduced with permission from the publisher Comingsoon.net and copyright owner Universal Studios.

The conceptual design of the nanometric hologram using the Sb_2_Te_3_ thin film is illustrated in Fig. 1. First, a binary phase-only hologram of the object is calculated by a computer (Supplementary Fig. 1). Then we record the binary hologram into the Sb_2_Te_3_ thin film through programmed multifocal spots created by the Debye diffraction method^22^,^23^ as shown in Fig. 1a. A home-built DLW system is used for the binary hologram fabrication (see Methods section). The binary holograms of the object images are first converted into a series of phase diagrams, which are uploaded onto the spatial light modulator in certain time orders. Then the binary holograms are printed into the Sb_2_Te_3_ thin film through synchronously moving the sample stage in the *xy*-plane. The number of focal-spots of the laser beam is 60 by 60 and the diameter of each spot is 2 μm. The size of the fabricated hologram is 3 × 3 mm^2^ and the number of pixels is 1,500 × 1,500. The laser beam fluence is kept at 0.1 J cm^−2^.

The upper inset in Fig. 1a displays two pixels ablated by the focused laser spots in the Sb_2_Te_3_ thin film. The area of the thin film without ablation leads to an enhanced phase shift due to the resonant cavity inside it. Based on this principle, a phase-only binary hologram is realized. Figure 1b shows the holographic imaging procedure by using a continuous-wave laser beam. Holographic images are reconstructed by illuminating the Sb_2_Te_3_ thin film with a laser beam and are captured by a colour CCD camera. The projection angle *θ* of the holographic image is designed to avoid the overlapping between the holographic image and the zero-order beam.

### Analysis of the multilayer structure

**Figure 2:**
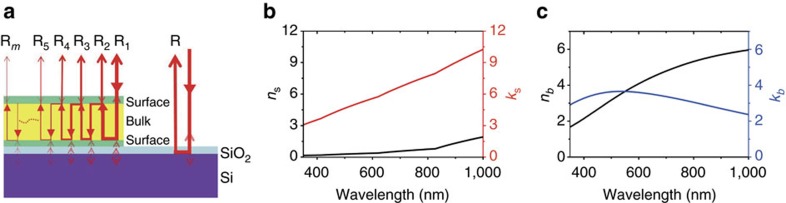
Physical mechanism of the Sb_2_Te_3_ thin film cavity. (**a**) Diagram of internal light multiple reflections in the resonant cavity of the Sb_2_Te_3_ thin film. The semi-transparent arrows indicate interface reflections. (**b**) The surface refractive index *n* and extinction coefficient *k* curves of the Sb_2_Te_3_ thin film as a function of wavelengths. (**c**) The bulk refractive index *n* and extinction coefficient *k* curves of the Sb_2_Te_3_ thin film as a function of wavelengths.

The multilayer structure of the Sb_2_Te_3_ thin film on a Si substrate is schematically shown in Fig. 2a. The dielectric bulk of the Sb_2_Te_3_ thin film is sandwiched within the two metallic surface layers. The refractive index (*n*) and extinction coefficient (*k*) of the surface layers and the bulk of the Sb_2_Te_3_ thin film are measured by using a spectroscopic ellipsometer (see Methods section). The measured wavelengths range from 350 to 1,000 nm. The measured data are fitted by using a B-spline model for the surface layers and a Tauc-Lorentz model for the bulk. The details of these models can be found in our previous work^21^.

Figure 2b shows the *n* and *k* curves of the surface layers of the Sb_2_Te_3_ thin film. Figure 2c shows the *n* and *k* curves of the bulk of the film. The value of bulk *n* agrees well with the previously reported values elsewhere^24^. Both *n* and *k* values are unequal in the bulk and the surface layers from visible to near-infrared wavelength ranges. With the unequal refractive indices, an Sb_2_Te_3_ thin film acts as an intrinsic optical resonant cavity. Two surface layers serve as two reflectors. The bulk behaves as an optical resonant cavity. Thus an incident light beam can be reflected multiple times between two surface layers and partially be confined in the bulk. The phase modulation of the reflected light beam from the resonant cavity can be enhanced.

**Figure 3:**
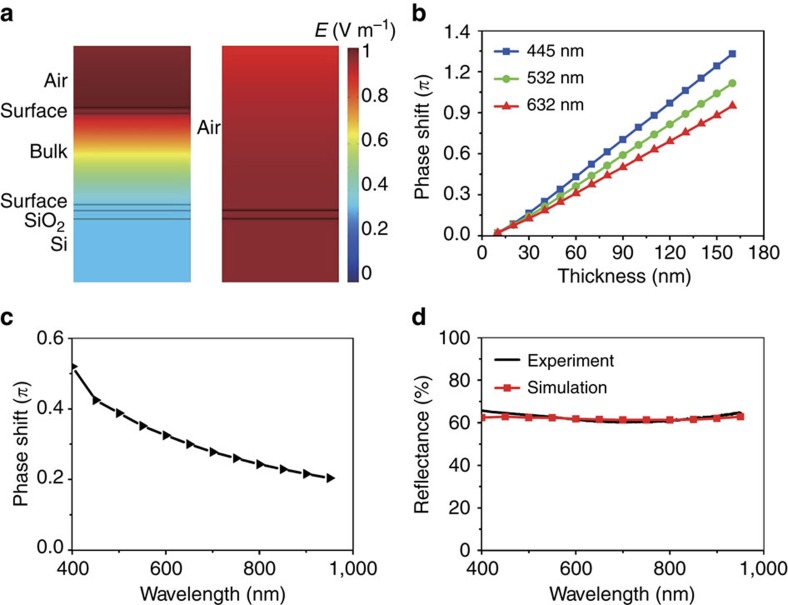
Numerical simulations of electromagnetic fields and phase shift. (**a**) Simulated electromagnetic field distributions in the Sb_2_Te_3_ thin film cavity and the SiO_2_/Si substrate under illumination with 632 nm light. (**b**) Simulated phase shift between the Sb_2_Te_3_ film and the SiO_2_/Si substrate as a function of the film thickness at different wavelengths. (**c**) Simulated phase shift between the 60 nm Sb_2_Te_3_ film and the SiO_2_/Si substrate as a function of wavelength. (**d**) Simulated and experimental reflectance of 60 nm Sb_2_Te_3_ thin films as a function of wavelength. The simulation shows good agreement with experiments.

The COMSOL Multiphysics modelling software is used to simulate the reflection and phase shift of the output light beams from the Sb_2_Te_3_ thin film and the Si substrate with a native SiO_2_ layer (−2.73 nm). The detailed theoretical calculation of the reflection and the phase shift can be found in Supplementary Note 1. The optical parameters for the simulation of the Sb_2_Te_3_ thin film can be obtained from Fig. 2b,c and the parameters for Si are obtained from the reference^25^. Figure 3a shows the simulated distributions of electromagnetic fields in the resonant cavity under illumination of the 632 nm laser beam and the electromagnetic fields uniformly distribute on the Si substrate without the Sb_2_Te_3_ thin film for comparison. Distinct enhancement of electromagnetic fields can be noticed in the resonant cavity.

The simulated phase shift (between the 60 nm Sb_2_Te_3_ thin films and the SiO_2_/Si substrate) as a function of the film thickness at the wavelengths 445, 532 and 632 nm light are shown in Fig. 3b. It is obvious that the phase shift monotonically increases when the thickness of the Sb_2_Te_3_ thin films increases. The phase shift is ∼0.5*π*, 0.4*π* and 0.3*π* at the wavelengths 445, 532 and 632 nm, respectively. Figure 3c shows the simulated phase shift between the Sb_2_Te_3_ thin film and SiO_2_/Si as a function of wavelength. It can be found that the phase shift decreases when the wavelength increases. These results demonstrate that Sb_2_Te_3_ thin films generate higher quality holographic images with shorter wavelengths. Figure 3d demonstrates the simulated and experimental reflectance of the 60 nm Sb_2_Te_3_ thin film as a function of wavelength. It can be found that the simulated reflectance agrees with experimental results very well. The simulated reflectance as a function of thin film thickness can be found in Supplementary Fig. 3a, and the reflectance of substrate can be found in Supplementary Fig. 3b.

### Optical characterization of nanometric holograms

Sb_2_Te_3_ has been both theoretically and experimentally confirmed as a topological insulator material^26^,^27^. In this work, the Sb_2_Te_3_ thin films are grown on Si wafers by using an atomic layer deposition (ALD) system. The ALD method is capable of growing nanometric films layer by layer on a large scale and at a relatively low temperature. The growth details of the Sb_2_Te_3_ thin films can be found in the Methods.

**Figure 4:**
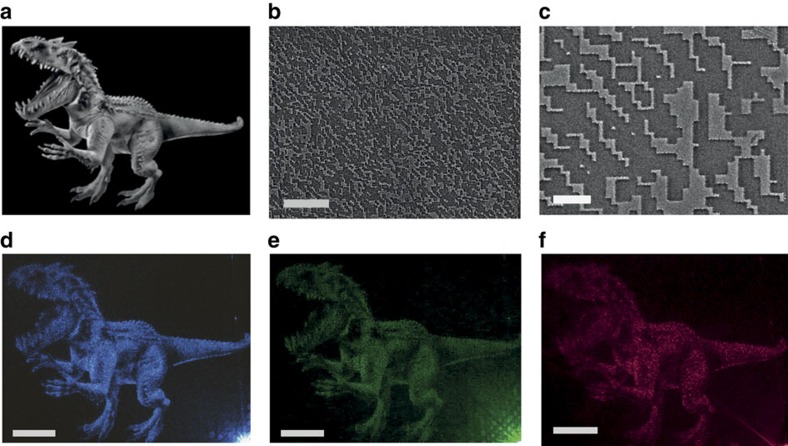
Nanometric Sb_2_Te_3_ thin film holograms and reconstructed images. (**a**) Original image of the dinosaur object. Note: this figure is not included under the article CC BY licence; Indominus Rex image is reproduced with permission from the publisher Comingsoon.net and copyright owner Universal Studios. (**b**,**c**) SEM images of the laser printed hologram patterns. The scale bar is 50 μm for **b** and 10 μm for **c**, respectively. (**d**–**f**) Holographic images captured by illuminating the nanometric holograms using 445, 532 and 632 nm continuous wavelaser beams. Scale bars for **d**–**f** are 1 mm.

Figure 4b,c show the scanning electron microscope (SEM) images of laser written hologram patterns on the Sb_2_Te_3_ thin films. With fs-laser ablation, the nanometric Sb_2_Te_3_ film is completely removed and the native SiO_2_ film is exposed to air. The Sb_2_Te_3_ film in the area without laser ablation results in a strong phase modulation due to multiple reflections in the optical cavity. However, the native SiO_2_ layer generates a weak phase modulation due to its small refractive index. Thus, two levels of phase modulations can be generated, leading to the formation of binary holograms.

The holographic images can be generated through the reconstruction of wavefronts of light with the phase information. By illuminating a laser beam on the surface of the topological insulator hologram, the holographic images are diffracted from it with a small projection angle of 10°. A full colour CCD was used to capture the reconstructed images. The laser beam power and its focal position are adjusted to obtain the optimal imaging quality. Figure 4a displays the original picture of dinosaur object. Figure 4d–f show the holographic images of the dinosaur from the 60 nm-thick hologram at different wavelengths. The signal-to-noise ratio, which is defined as the ratio of the peak intensity in the reconstructed image to the s.d. of the background noise^10^, can be utilized to evaluate the quality of the reconstructed image. The background noise is taken from an area of 120 × 120 pixels beside the image. The signal-to-noise ratios for the reconstructed images from Fig. 4d–f are 39, 33 and 29, respectively. The reconstructed images demonstrate a higher quality at shorter wavelengths due to the larger phase modulation.

The measured conversion efficiency of the 60 nm-thick hologram is approximately 1.6, 1.5 and 1.35% at the wavelengths 445, 532 and 632 nm, respectively. The conversion efficiency is defined as the ratio of the power of the holographic image and the input power. In comparison, the efficiency of topological insulator holograms is lower than dielectric metasurface holograms. One main reason is that the hologram we have used is a binary one which causes more noises than multiple-level holograms. Another reason is that the phase shift for a 60 nm hologram (0–*π*/2) is still smaller than a metasurface modulated phase shift (0–2*π*). Through increasing the thickness and the phase modulation levels, the topological insulator hologram efficiency can be further improved. In addition, the light absorption in the Sb_2_Te_3_ thin film is higher than dielectric metasurface in the visible range. The efficiency of topological insulator holograms can be improved through using highly insulating topological insulator materials with a higher bulk refractive index.

## Discussion

The performance of the nanometric holograms is determined by several parameters. First, in the nanofabrication process, the power density of the fs-laser beam affects the hologram morphology and resolution. In our experiments, the laser beam fluence of 0.1 J cm^−2^ is optimal for obtaining high quality hologram patterns and holographic images. Second, the thickness of the Sb_2_Te_3_ thin films plays a key role in the quality and efficiency of holographic images. In principle, the thicker holograms generate a larger phase shift and thus higher quality holographic images. Furthermore, the images noise is lower for thicker holograms. These can be found through comparing the 60 nm thick hologram with a 25 nm-thick hologram in Supplementary Fig. 4c. Third, in the holographic imaging process, the laser wavelengths affect the holographic images and the hologram efficiency. We have found that the shorter wavelength produces the higher efficiency due to the larger phase shift. However, the light absorption is enhanced in the ultraviolet frequency range, which limits the further improvement of the hologram quality and efficiency. Therefore, to achieve the best holographic image, these parameters need to be balanced.

Compared with the fabrication methods of metasurface holograms, the DLW method does not require the complicated substrate treatment and the mask preparation process. Hence, the DLW fabricated nanometric holograms are more suitable for large-scale practical applications. They are promising to be integrated into flat electronic devices for optical imaging, data storage and security. They can also be used as anti-fake stamps for credit cards and currencies. In addition, with the unique function of enhancing phase shifts, a topological insulator cavity can also be used to design nanometric photo-detectors and lenses.

## Methods

### ALD growth of Sb_2_Te_3_ thin films

The chemical equation for synthesizing Sb_2_Te_3_ is Sb(OC_2_H_5_)_3_+3[(CH_3_)_3_Si]_2_Te→Sb_2_Te_3_+6(CH_3_)_3_Si-OC_2_H_5_ (ref. 28). The injection/purge times for the precursors of Sb(OC_2_H_5_)_3_ and 3[(CH_3_)_3_Si]_2_Te were 0.75 s/9 s and 0.1 s/9 s, respectively. As Sb(OC_2_H_5_)_3_ was difficult to gasify, an Ar gas booster was used on the cylinder to promote the gasification process. The precursor transport temperature was kept at 150 °C. The substrate temperature in the deposition process was kept at 70 °C, which was optimal for a maximum growth rate. The chamber vacuum was 2 × 10^−2^ Torr. The growth rate was 0.01 nm per cycle.

The X-ray diffraction patterns (Supplementary Fig. 2a) identify that the Sb_2_Te_3_ thin film is highly crystalline and grown along the *c* axis. The Raman spectrum (Supplementary Fig. 2b) confirms that the composition of the thin film is Sb_2_Te_3_. The Atomic Force Microscopy (Supplementary Fig. 2c) demonstrates the surface morphology of the thin films. The measured bulk band gap of the Sb_2_Te_3_ thin films is 0.15 eV (Supplementary Fig. 3c). These characterizations reveal the high quality of the ALD grown Sb_2_Te_3_ thin film.

### Direct Laser writing system

A femtosecond-pulsed laser beam at a wavelength of 800 nm (Maitai, repetition rate: 10 KHz and pulse width: 100 fs) was employed for the writing of hologram patterns. An objective with numerical aperture of 0.7 was used to focus the pulsed laser beam onto the surface of the Sb_2_Te_3_ thin film. The average laser beam fluence was adjusted by an ND filter and was kept at 0.1 Jcm^−2^ in the fabricating process. The holographic patterns were achieved by laterally translating the 3D translation stage across the focal plane.

### Optical measurements

The refractive index and extinction coefficient were measured using a multiple-angle spectroscopic ellipsometer (J.A. Woollam Co. M-2000). The thickness of the Sb_2_Te_3_ thin film was determined through fitting the data from ellipsometer measurements. The thickness of the surface layer was 1.5 nm, and the thickness remains constant when the total thickness of the thin film increases. The reflection spectroscopy was measured using an ultraviolet–visible spectrophotometer.

### Data availability

All data used to obtain the conclusions in this paper are available in the paper and/or the Supplementary Information. Other data may be requested from the authors.

## Additional information

**How to cite this article:** Yue, Z. *et al*. Nanometric holograms based on a topological insulator material. *Nat. Commun.* **8,** 15354 doi: 10.1038/ncomms15354 (2017).

**Publisher's note**: Springer Nature remains neutral with regard to jurisdictional claims in published maps and institutional affiliations.

## Supplementary Material

Supplementary Information Supplementary Figures, Supplementary Note and Supplementary References.

## References

[b1] Gabor D. A new microscopic principle. Nature 161, 777–778 (1948). 18860291 10.1038/161777a0

[b2] Li X. . Athermally photoreduced graphene oxides for three-dimensional holographic images. Nat. Commun. 6, 6984 (2015). 25901676 10.1038/ncomms7984 PMC4421811

[b3] Ye W. . Spin and wavelength multiplexed nonlinear metasurface holography. Nat. Commun. 7, 11930 (2016). 27306147 10.1038/ncomms11930 PMC4912630

[b4] Brown B. R. & Lohmann A. W. Complex spatial filtering with binary masks. Appl. Opt. 5, 967–969 (1966). 20048989 10.1364/AO.5.000967

[b5] Slinger C., Cameron C. & Stanley M. Computer-generated holography as a generic display technology. Computer 38, 46–53 (2005).

[b6] Xue G. . Multiplexing encoding method for full-color dynamic 3D holographic display. Opt. Express 22, 18473–18482 (2014). 25089466 10.1364/OE.22.018473

[b7] Yu N. & Capasso F. Flat optics with designer metasurfaces. Nat. Mater. 13, 139–150 (2014). 24452357 10.1038/nmat3839

[b8] Kildishev A. V., Boltasseva A. & Shalaev V. M. Planar photonics with metasurfaces. Science 339, 1232009 (2013). 23493714 10.1126/science.1232009

[b9] Ren H., Li X., Zhang Q. & Gu M. On-chip noninterference angular momentum multiplexing of broadband light. Science 352, 805–809 (2016). 27056843 10.1126/science.aaf1112

[b10] Ni X. J., Kildishev A. V. & Shalaev V. M. Metasurface holograms for visible light. Nat. Commun. 4, 2807 (2013).

[b11] Huang L. . Three-dimensional optical holography using a plasmonic metasurface. Nat. Commun. 4, 2808 (2013).

[b12] Zheng G. . Metasurface holograms reaching 80% efficiency. Nat. Nanotechnol. 10, 308–312 (2015). 25705870 10.1038/nnano.2015.2

[b13] Khorasaninejad M., Ambrosio A., Kanhaiya P. & Capasso F. Broadband and chiral binary dielectric meta-holograms. Sci. Adv. 2, e1501258 (2016). 27386518 10.1126/sciadv.1501258 PMC4928906

[b14] Bonaccorso F., Sun Z., Hasan T. & Ferrari A. C. Graphene photonics and optoelectronics. Nat. Photon. 4, 611–622 (2010).

[b15] Wang Q. H., Kalantar-Zadeh K., Kis A., Coleman J. N. & Strano M. S. Electronics and optoelectronics of two-dimensional transition metal dichalcogenides. Nat. Nanotechnol. 7, 699–712 (2012). 23132225 10.1038/nnano.2012.193

[b16] Hasan M. Z. & Kane C. L. Colloquium: topological insulators. Rev. Mod. Phys. 82, 3045–3067 (2010).

[b17] Qi X.-L. & Zhang S.-C. Topological insulators and superconductors. Rev. Mod. Phys. 83, 1057–1110 (2011).

[b18] Peng H. . Topological insulator nanostructures for near-infrared transparent flexible electrodes. Nat. Chem. 4, 281–286 (2012). 22437712 10.1038/nchem.1277

[b19] Di Pietro P. . Observation of Dirac plasmons in a topological insulator. Nat. Nanotechnol. 8, 556–560 (2013). 23872838 10.1038/nnano.2013.134

[b20] Ou J.-Y. . Ultraviolet and visible range plasmonics in the topological insulator Bi_1.5_Sb_0.5_Te_1.8_Se_1.2_. Nat. Commun. 5, 5139 (2014). 25295413 10.1038/ncomms6139

[b21] Yue Z., Cai B., Wang L., Wang X. & Gu M. Intrinsically core-shell plasmonic dielectric nanostructures with ultrahigh refractive index. Sci. Adv. 2, e1501536 (2016). 27051869 10.1126/sciadv.1501536 PMC4820380

[b22] Lin H., Jia B. & Gu M. Dynamic generation of Debye diffraction-limited multifocal arrays for direct laser printing nanofabrication. Opt. Lett. 36, 406–408 (2011). 21283205 10.1364/OL.36.000406

[b23] Gu M. Advanced Optical Imaging Theory Springer Science & Business Media (2000).

[b24] Kuwahara M. . Approach for measuring complex refractive index of molten Sb2Te3 by spectroscopic ellipsometry. Appl. Phys. Lett. 100, 101910 (2012).

[b25] Green M. A. & Keevers M. J. Optical properties of intrinsic silicon at 300 K. Prog. Photovolt. 3, 189–192 (1995).

[b26] Zhang H. . Topological insulators in Bi_2_Se_3_, Bi_2_Te_3_ and Sb_2_Te_3_ with a single Dirac cone on the surface. Nat. Phys. 5, 438–442 (2009).

[b27] Zhang J. . Band structure engineering in (Bi_*1−x*_Sb_*x*_)_2_Te_3_ ternary topological insulators. Nat. Commun. 2, 574 (2011). 22146393 10.1038/ncomms1588

[b28] Eom T. . Conformal formation of (GeTe_2_)(1–*x*)(Sb_2_Te_3_)_*x*_ layers by atomic layer deposition for nanoscale phase change memories. Chem. Mater. 24, 2099–2110 (2012).

