# Supplementary material for: Nanometric holograms based on a topological insulator material
Source: Nat Commun. 2017 May 18;8:15354. doi: 10.1038/ncomms15354 (PMC5454374; doi:10.1038/ncomms15354)
Supplement: Supplementary Information — Supplementary Figures, Supplementary Note and Supplementary References. [file ncomms15354-s1.pdf]

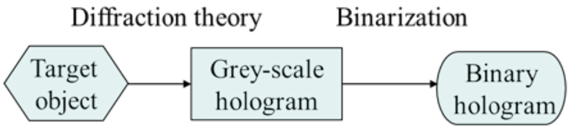

Supplementary Figure 1: Computing methods of a phase-only binary hologram. The grey-scale hologram of an object can be obtained based on the point source method[1] or the polygon-based method[2]. For the binarization of the holograms, we can choose the median number of it as the threshold, and then convert all its numbers to 0 or π.

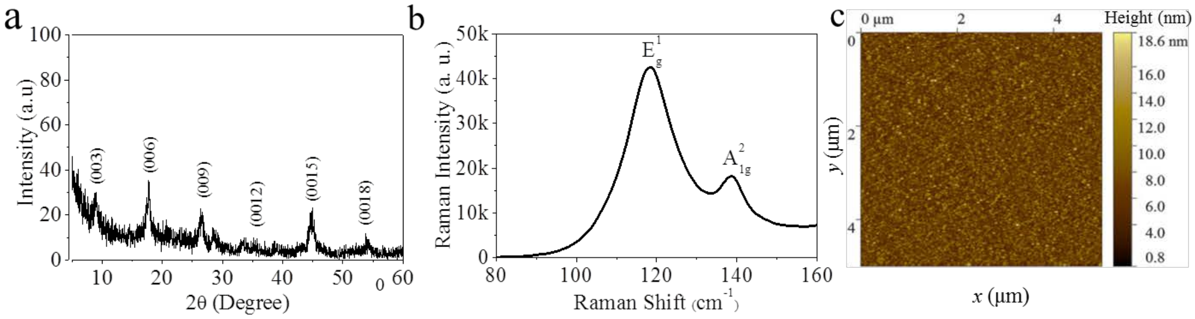

Supplementary Figure 2: Material characterization. (a) XRD patterns of the Sb_2Te_3 thin film grown on the Si substrate. The Sb_2Te_3 thin film is highly crystalline and has a preferred orientation with respect to the *c*-axis. The diffraction peaks can be indexed on the basis of the structure of rhombohedral Sb_2Te_3. (b) Raman spectroscopy of the Sb_2Te_3 thin film on Si. Based on the Raman spectra, we confirm that the Sb-Te thin film have Sb_2Te_3 stoichiometry. (c) AFM (Bruker DI 3100) image of the Sb_2Te_3 thin films on Si.

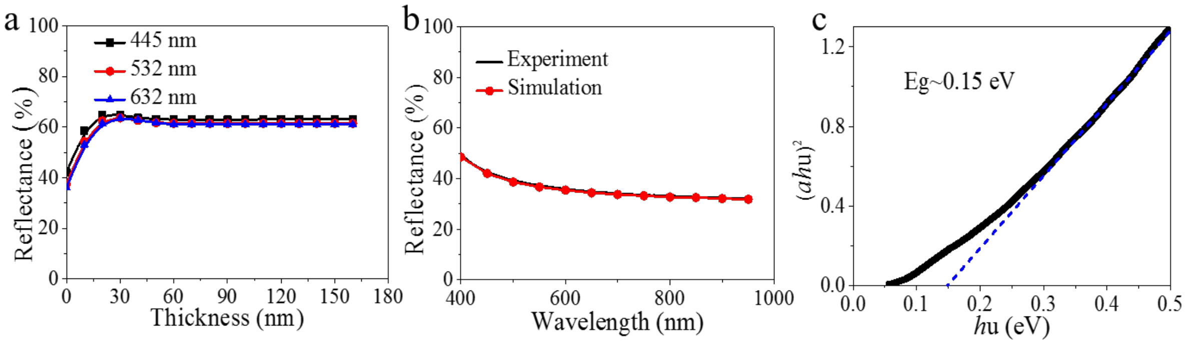

Supplementary Figure 3: Reflectance and absorption. (a) The simulated reflectance as a function of the thickness of Sb_2Te_3 thin films at different wavelengths. The reflection keeps nearly constant in the thick film due to the disappearance of both light transmission and absorption by the Si substrate. Nearly all the light beam is reflected by the thin film, which keeps invariant. (b) Simulated and experimental reflectance of the SiO_2/Si substrate as a

function of wavelengths. The simulation and experiment fit very well. (c) Tauc plot[3] of the measured absorption spectra of the 60 nm-thick Sb_2Te_3 film. The obtained bulk band gap is ~0.15 eV, which is much higher than the room-temperature energy of 0.026 eV.

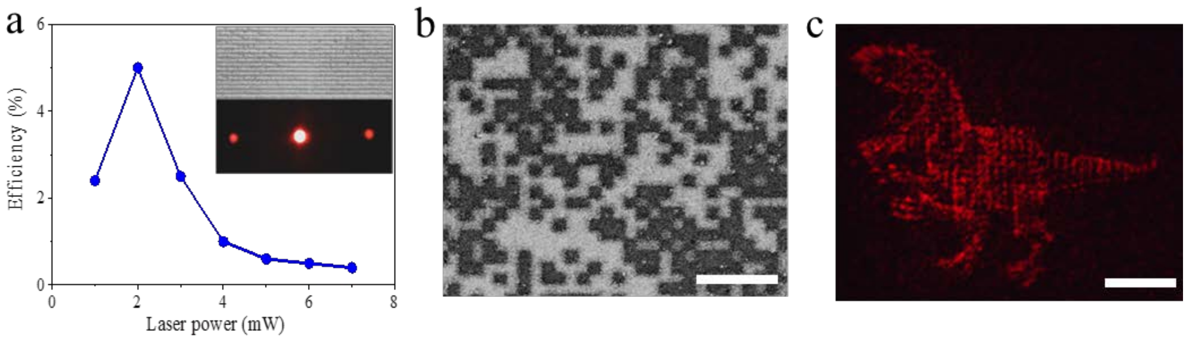

Supplementary Figure 4: 25 nm thick Sb_2Te_3 thin film hologram. (a) Diffraction efficiency of the laser fabrication method[4]. The insets are the grating image and 0th and ±1st diffraction orders. The grating has a thickness of 25 nm, a line width of 2 μm and a period of 4 μm. The purpose of measuring the Sb_2Te_3 diffraction grating is to determine the diffraction efficiency of the laser ablation method. (b) SEM images of 25 nm hologram patterns. (c) Holographic images of dinosaur from the 25 nm Sb_2Te_3 holograms under illumination of a 632 nm laser beam. Note: This figure is not included under the article CC BY licence; Indominus Rex image is reproduced with permission from the publisher Comingsoon.net and copyright owner Universal Studios.

Supplementary Note 1: Theoretical calculation of phase shift and reflectance

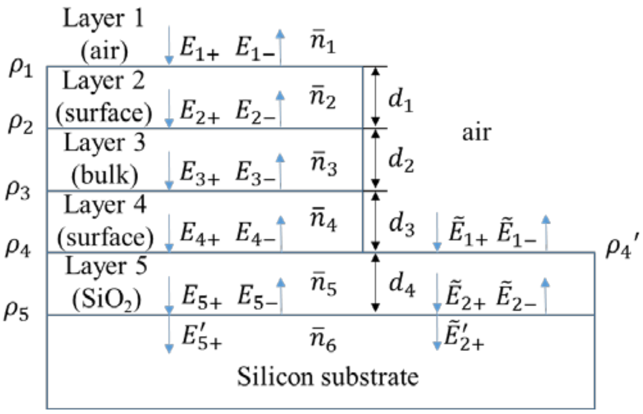

Supplementary Figure 5: Multi-reflections in multilayer structure with Sb_2Te_3 and without Sb_2Te_3

Here, we first study the multilayer structure of air/surface layer/bulk layer/surface layer/SiO_2/Si substrate, as shown in the left half part of Supplementary Figure 5. The reflection coefficients ρ_j from the top of each interface is defined as

$$\rho_j = \frac{\bar{n}_j - \bar{n}_{j+1}}{\bar{n}_j + \bar{n}_{j+1}}, j = 1,2,3,4,5 \quad (1)$$

where $\bar{n}_j = n_j + ik_j$, n_j is the refractive index and k_j is the extinction coefficient of the ith layer. The incident and reflected electric fields are considered at the top of each interface and the overall reflection response can be expressed as $\Gamma = E_{1-}/E_{1+}$[5]. The downward/upward fields (E_{j+}, E_{j-}) at the top of interface i are related to those at the top of interface $j+1$ by

$$\begin{bmatrix} E_{j+} \\ E_{j-} \end{bmatrix} = \frac{1}{\tau_j} \begin{bmatrix} e^{-i\sigma_j} & \rho_j e^{i\sigma_j} \\ \rho_j e^{-i\sigma_j} & e^{i\sigma_j} \end{bmatrix} \begin{bmatrix} E_{(j+1)+} \\ E_{(j+1)-} \end{bmatrix}, (j = 1,2,3,4) \quad (2)$$

where $\tau_j = 1 + \rho_j$, σ_j is the phase shift from interface j to interface $j+1$, $\sigma_i = 2\pi d_j \bar{n}_{j+1}/\lambda$. d_j is the thickness of $(j+1)$th layer and λ is the wavelength of light. Assume that no upward waves in the Silicon substrate. These recursions are initialized at the 5th interface as follows:

$$\begin{bmatrix} E_{5+} \\ E_{5-} \end{bmatrix} = \frac{1}{\tau_5} \begin{bmatrix} 1 & \rho_5 \\ \rho_5 & 1 \end{bmatrix} \begin{bmatrix} E'_{5+} \\ 0 \end{bmatrix}. \quad (3)$$

Then the overall reflection response can be calculated by $\Gamma = E_{1-}/E_{1+} = r\exp(i\varphi)$, where r is the reflection coefficient of the multilayer structure and φ is the phase shift generated by the multilayer structure.

In the same way, the overall reflection response of the air/SiO_2/Si substrate can also be calculated, which can be given as $\tilde{\Gamma} = \tilde{E}_{1-}/\tilde{E}_{1+} = \tilde{r}\exp(i\tilde{\varphi})$, where $\tilde{r}$ is the reflection coefficient and $\tilde{\varphi}$ is the phase shift caused by the three-layer structure. Finally, the phase shift between Sb_2Te_3 film and SiO_2/Si substrate can be obtained by

$$\Delta\varphi = \varphi - \tilde{\varphi} - 4\pi n_1(d_1 + d_2 + d_3)/\lambda. \quad (4)$$

Matlab was utilized in the realization of numerical modelling, which outputted the results in accordance with that calculated by Comsol software.

Supplementary References

1. Gao C, Liu J, Li X, Xue G, Jia J, Wang Y. Accurate compressed look up table method for CGH in 3D holographic display. *Opt Express* 23, 33194-33204 (2015).

2. Pan Y, Wang Y, Liu J, Li X, Jia J. Fast polygon-based method for calculating computer-generated holograms in three-dimensional display. *Applied Optics* 52, A290-A299 (2013).

3. Tauc J. Optical properties and electronic structure of amorphous Ge and Si. *Materials Research Bulletin* 3, 37-46 (1968).

4. Li X, et al. Athermally photoreduced graphene oxides for three-dimensional holographic images. *Nat Commun* 6, 6984 (2015).

5. M. Abdul-Gader Jafar M., Comprehensive formulations for the total normal-incidence optical reflectance and transmittance of thin films laid on thick substrates, *European International Journal of Science and Technology*, 2, 214 (2013).
